# The pre-mRNA splicing factor ZOP1 contributes to the regulation of plant immunity

**DOI:** 10.1093/plphys/kiaf351

**Published:** 2025-08-06

**Authors:** Yiran Wang, Weijie Huang, Pingyu Zhang, Xin Li, Yuelin Zhang

**Affiliations:** Department of Botany, University of British Columbia, Vancouver, BC V6T 1Z4, Canada; Department of Botany, University of British Columbia, Vancouver, BC V6T 1Z4, Canada; The College of Life Sciences, Sichuan University, Chengdu, Sichuan 610064, China; Department of Botany, University of British Columbia, Vancouver, BC V6T 1Z4, Canada; Michael Smith Laboratories, University of British Columbia, Vancouver, BC V6T 1Z4, Canada; Department of Botany, University of British Columbia, Vancouver, BC V6T 1Z4, Canada; The College of Life Sciences, Sichuan University, Chengdu, Sichuan 610064, China

## Abstract

The *Arabidopsis* (*Arabidopsis thaliana)* receptor-like protein SUPPRESSOR OF NPR1-1, CONSTITUTIVE 2 (SNC2) confers basal resistance against pathogens. The transcription factor CALMODULIN-BINDING PROTEIN 60 g (CBP60g) and its homolog SYSTEMIC ACQUIRED RESISTANCE DEFICIENT 1 (SARD1) define two parallel pathways downstream of SNC2. Here, we report the identification and characterization of ZINC-FINGER AND OCRE DOMAIN-CONTAINING PROTEIN 1 (ZOP1), a pre-mRNA splicing factor that functions as a positive regulator of SNC2-mediated immunity. ZOP1 functions immediately downstream of SNC2 before the defense signals branch to CBP60g and SARD1. Loss-of-function mutation in *ZOP1* suppresses *snc2-1D*-mediated autoimmunity, while *zop1* single mutants exhibit compromised resistance against pathogens. RNA-sequencing analysis revealed that many defense response genes are differentially expressed or have altered splicing patterns in the *zop1* mutant. The genes downregulated in the *zop1* mutant include *BDA1* and *RLP23*, which encode two key regulators of plant defense. Overall, our study suggests that ZOP1 contributes to the regulation of plant immunity.

## Teaser

The pre-mRNA splicing factor ZOP1 contributes to the regulation of SNC2-mediated immunity by indirectly modulating the transcript level of *BDA1*.

## Introduction

Plants rely on their innate immune system to perceive and ward off pathogen threats. They utilize cell surface localized pattern-recognition receptors (PRRs) to sense the conserved pathogen-associated molecular patterns (PAMPs), leading to the activation of pattern triggered immunity (PTI) (Boller and Felix 2009). Adapted pathogens can deliver effectors to interfere with PTI, leading to effector triggered susceptibility (Jones and Dangl 2006). In turn, plants have evolved intracellular resistance (R) proteins to recognize specific pathogen effectors and activate robust effector triggered immunity, which usually leads to localized hypersensitive cell death response (Belkhadir et al. 2004).

Some PRRs belong to the receptor-like protein (RLP) family. A typical RLP has an extracellular leucine-rich repeat domain, a transmembrane motif, and a tiny cytoplasmatic tail (Zipfel 2014). *Arabidopsis* (*Arabidopsis thaliana)* RLP SUPPRESSOR OF NPR1-1, CONSTITUTIVE 2 (SNC2) plays an essential role in basal resistance and PTI (Yang et al. 2012; Zhang et al. 2010b). Loss of SNC2 results in compromised resistance against pathogenic bacteria *Pseudomonas syringae* pv *tomato* (*Pst*) DC3000 as well as nonpathogenic bacteria *Pst* DC3000 *hrcC^-^*. Conversely, a gain-of-function mutation in the conserved GXXXG motif of the SNC2 transmembrane domain leads to constitutively activated defense responses. This *snc2-1D* mutant has smaller size, accumulates high levels of defense hormone salicylic acid (SA), constitutively expresses *PATHOGENESIS-RELATED* (*PR*) genes, and shows enhanced pathogen resistance to oomycete pathogen *Hyaloperonospora arabidopsidis* (*Hpa*) Noco2 (Zhang et al. 2010b).

The plant-specific transcription factor CALMODULIN-BINDING PROTEIN 60 g (CBP60g) and SYSTEMIC ACQUIRED RESISTANCE DEFICIENT 1 (SARD1) are two master transcription factors in plant immunity (Zhang et al. 2010a). They function downstream of SNC2 and contribute to the autoimmunity of *snc2-1D* (Sun et al. 2015). The autoimmune responses of *snc2-1D* were partially blocked in *sard1-1 snc2-1D* and *cbp60g-1 snc2-1D* double mutants and almost completely abolished in the *sard1-1 cbp60g-1 snc2-1D* triple mutant. The dwarfism of *snc2-1D* was slightly attenuated in *sard1-1 snc2-1D* and *cbp60g-1 snc2-1D*, whereas *cbp60g-1 sard1-1 snc2-1D* was wild-type-like. Therefore, SARD1 and CBP60g function in parallel and define two independent pathways that orchestrate SNC2-mediated immunity (Sun et al. 2015).

Alternative splicing of mRNA precursors expands the transcript diversity in eukaryotes. Pre-mRNA splicing is performed by the spliceosome, a dynamic protein complex containing more than 100 proteins and 5 small nuclear ribonucleoprotein particles (Shang et al. 2017). In *Arabidopsis*, many splicing factors have been identified. Some of them were found to regulate plant abiotic stress responses. As examples, the splicing factor STABILIZED 1 (STA1) and RNA-DIRECTED DNA METHYLATION 16 (RDM16) are regulators of plant responses to high salinity, extreme temperatures, and drought stress (Lee et al. 2006; Huang et al. 2013; Zhang et al. 2013; Du et al. 2015; Kim et al. 2017). Some splicing factors such as SERINE/ARGININE-RICH 45 (SR45), GLYCINE-RICH RNA-BINDING PROTEIN 7 (GRP7), SUPPRESSOR OF ABI3-5 (SUA), and REQUIRED FOR SNC4-1D 2 (RSN2) were also shown to regulate plant immunity (Hackmann et al. 2014; Zhang et al. 2014; Chen et al. 2019). As a component of the spliceosome, ZINC-FINGER AND OCRE DOMAIN-CONTAINING PROTEIN 1 (ZOP1) was reported to function in pre-mRNA splicing, transcriptional gene silencing, and cold stress response (Zhang et al. 2013; Du et al. 2015). However, its role in plant immunity was not explored previously.

To search for immune regulators of *snc2-1D*-mediated immunity, a forward genetic screen was performed in the *sard1-1 snc2-1D* background. Here, we report the identification and characterization of suppressor 116-1. The 116-1 mutant contains a mutation in *ZOP1.* Further analysis showed that ZOP1 indirectly modulates the transcript level of *BDA1* (for Bian Da; “becoming big” in Chinese), a key component downstream of SNC2. Transcriptome analysis revealed that a large number of defense-related genes are differentially expressed or alternatively spliced in *zop1* mutant plants.

## Results

### Identification and characterization of suppressor mutant 116-1

**Figure 1.**
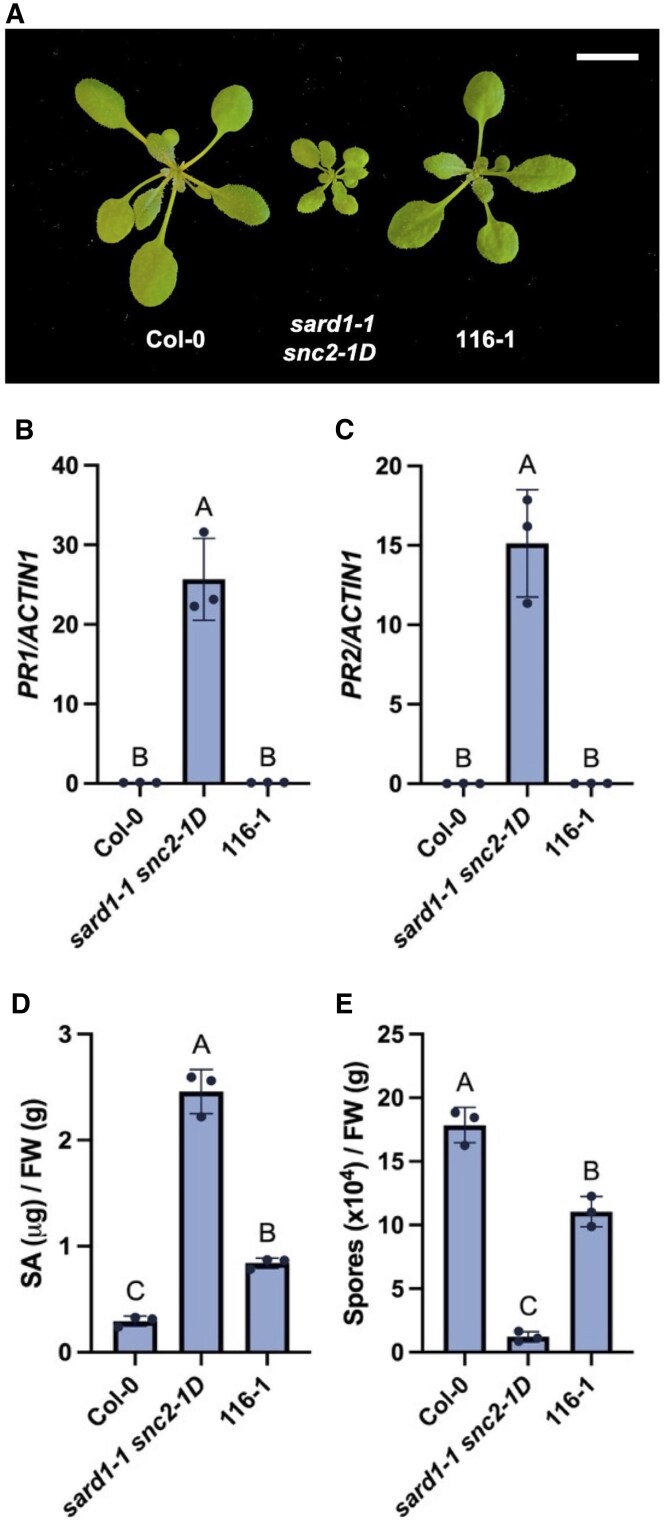
Identification and characterization of the *sard1-1 snc2-1D* suppressor mutant 116-1. **A)** Morphology of 3-week-old soil-grown plants of the indicated genotypes under long-day condition. Scale bar is 1 cm. **B** and **C)** Relative expression levels of *PR1*  **(B)** and *PR2*  **(C)** in the indicated genotypes. Transcript levels were normalized with *ACTIN1*. **D)** Free SA levels in the indicated genotypes. **E)** Growth of *Hpa* Noco2 conidiospores on the indicated genotypes. For **(B)** to **(E)**, error bars represent standard deviations. Letters indicate statistical differences (*P* < 0.05, one-way ANOVA; *n* = 3). FW, fresh weight. All experiments were repeated twice with similar results.

To search for immune regulators of *snc2-1D*-mediated immunity, a forward genetic screen was performed to identify mutants with suppressed autoimmunity of *sard1-1 snc2-1D*. Considering the trade-off between plant immunity and growth (Huot et al. 2014), the mutagenized population was screened for mutants with larger size than *sard1-1 snc2-1D*. 116-1 was one of the suppressor mutants recovered. As shown in Fig. 1A, 116-1 has an intermediate size between the wild-type Columbia (Col-0) and *sard1-1 snc2-1D*. It displayed reduced expression of the defense marker gene *PR1* and *PR2* compared to the *sard1-1 snc2-1D* mutant (Fig. 1, B and C). *sard1-1 snc2-1D* was previously reported to accumulate higher SA (Sun et al. 2015), and the SA level in 116-1 was considerably lower than in *sard1-1 snc2-1D* (Fig. 1D). The mutant 116-1 also supported more growth of the virulent oomycete pathogen *Hpa* Noco2 compared to *sard1-1 snc2-1D* (Fig. 1E). Thus, 116-1 shows suppression of all aspects of the autoimmune phenotypes of *sard1-1 snc2-1D*.

### Mapping-by-sequencing of 116-1

**Figure 2.**
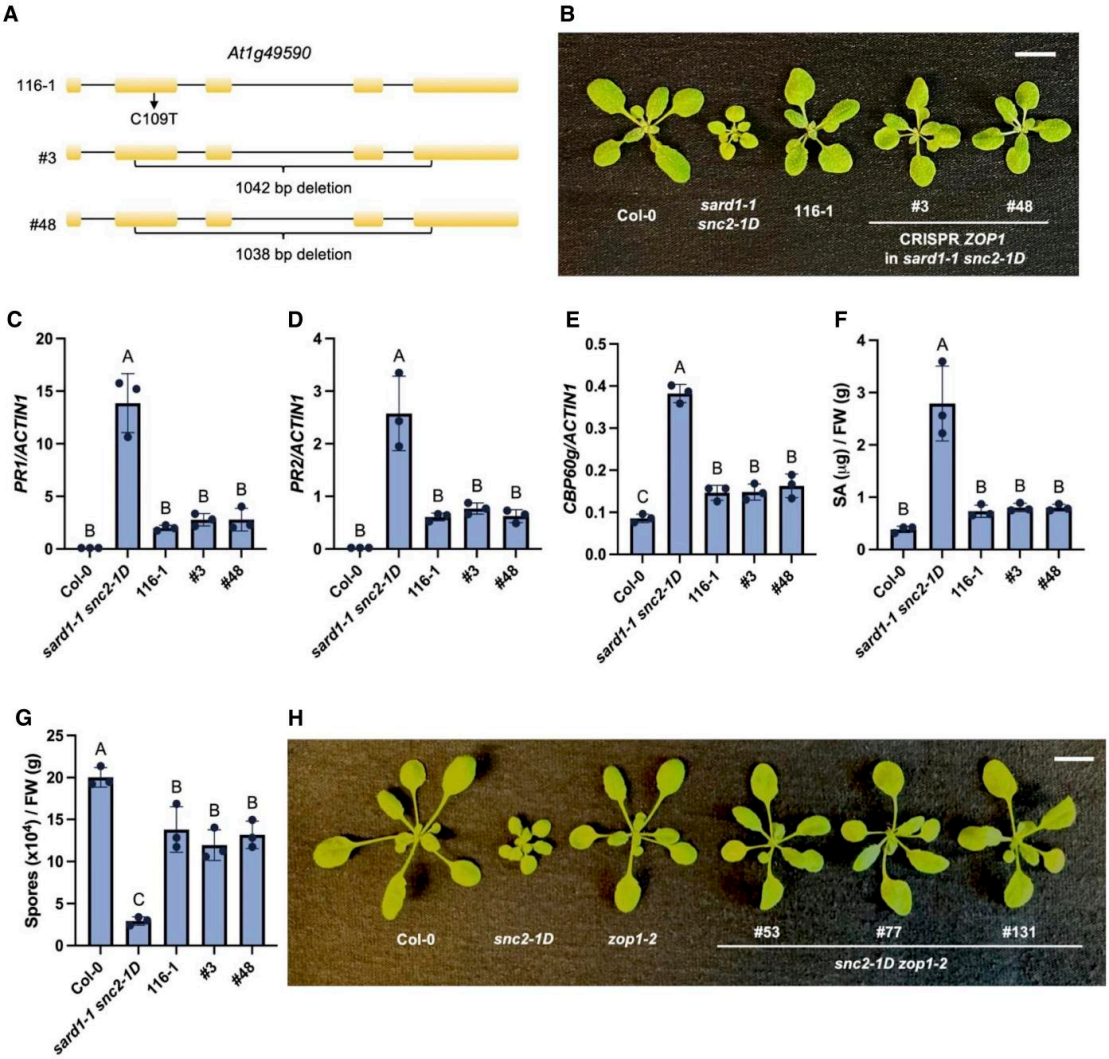
CRISPR deletion mutants of *ZOP1* suppress the autoimmunity of *sard1-1 snc2-1D*. **A)** Mutations in *ZOP1* (*At1g49590)*. 116-1 carries a C to T substitution in *At1g49590*, leading to a premature stop codon (top). #3 and #48 are two independent CRISPR deletion lines of *ZOP1* in the *sard1-1 snc2-1D* background (bottom). **B)** Morphology of 24-day-old soil-grown plants of the indicated genotypes under long-day condition. Scale bar is 1 cm. **C** to **E)** Expression levels of *PR1*  **(C)**, *PR2*  **(D)** and *CBP60g*  **(E)** in the indicated genotypes as normalized by *ACTIN1*. **F)** Free SA levels in the indicated genotypes. **G)** Growth of *Hpa* Noco2 conidiospores on the indicated genotypes. **H)** Morphologies of 21-day-old soil-grown plants of the indicated genotypes under long-day condition. Scale bar is 1 cm. For **(C)** to **(G)**, error bars represent standard deviations. Letters indicate statistical differences (*P* < 0.05, one-way ANOVA; *n* = 3). FW, fresh weight. All experiments were repeated twice with similar results.

When 116-1 was backcrossed to *sard1-1 snc2-1D*, the resulting F1 plants appeared *sard1-1 snc2-1D* like, indicating that 116-1 carries a recessive mutation (Supplementary Fig. S1A). In F2, a 130:39 segregation of suppressor-like and *sard1-1 snc2-1D*-like plants was observed, agreeing with the expected 3:1 ratio for a single recessive nuclear mutation (χ^2^ = 0.3521; *P*-value = 0.5637). Leaf tissue from the 36 confirmed homozygous F3 populations displayed no segregation of plants with *sard1-1 snc2-1D*-like phenotype was collected for DNA extraction and whole-genome sequencing (WGS). Upon data analysis, a linkage region was identified on Chromosome 1 (Supplementary Fig. S1B). In this region, one of the candidate genes *At1g49590* (encoding ZOP1) carries a C109T mutation located in the second exon, leading to a premature stop codon (Fig. 2A).

### Loss of *ZOP1* suppresses the autoimmunity of *sard1-1 snc2-1D*

To determine whether the mutation identified in *ZOP1* is responsible for suppressing *sard1-1 snc2-1D*, a CRISPR/Cas9 construct targeting *ZOP1* was generated and transformed into the *sard1-1 snc2-1D* background. Two independent CRISPR lines with large deletions in *ZOP1* displayed a larger plant size (Fig. 2B and Supplementary Fig. S2). The constitutive expression of *PR1*, *PR2*, and *CBP60g* of *sard1-1 snc2-1D* was largely suppressed in these CRISPR lines (Fig. 2, C to E). The elevated SA accumulation as well as enhanced disease resistance against *Hpa* Noco2 in *sard1-1 snc2-1D* was abolished in the CRISPR deletion lines (Fig. 2, F and G). Thus, we conclude that loss-of-function mutations in *ZOP1* suppress *sard1-1 snc2-1D*.

Further, when a construct expressing *ZOP1* driven by its native promoter, *pZOP1::ZOP1-3HA*, was transformed into 116-1, the plant size of transgenic lines was reverted back to that as *sard1-1 snc2-1D* (Supplementary Fig. S3A). Also, the transgenic lines restore the enhanced disease resistance against *Hpa* Noco2 (Supplementary Fig. S3B). These data further support the hypothesis that the mutation in *ZOP1* in 116-1 causes the autoimmunity of *sard1-1 snc2-1D.*

CBP60g and SARD1 define two parallel pathways downstream of SNC2 (Sun et al. 2015). By crossing the *zop1* CRISPR deletion allele *zop1-2* with *snc2-1D* mutant, homozygous *snc2-1D zop1-2* lines were obtained. As shown in Fig. 2H, the dwarfed morphology of *snc2-1D* was largely suppressed by the loss of *ZOP1*. Furthermore, loss of *ZOP1* in *cbp60g-1 snc2-1D* also suppressed the small stature of *cbp60g-1 snc2-1D* (Supplementary Fig. S4). Taken together, ZOP1 most likely functions upstream of both CBP60g-dependent and SARD1-dependent pathways in SNC2-mediated plant immunity.

### *zop1* single mutants exhibit compromised resistance against pathogen

**Figure 3.**
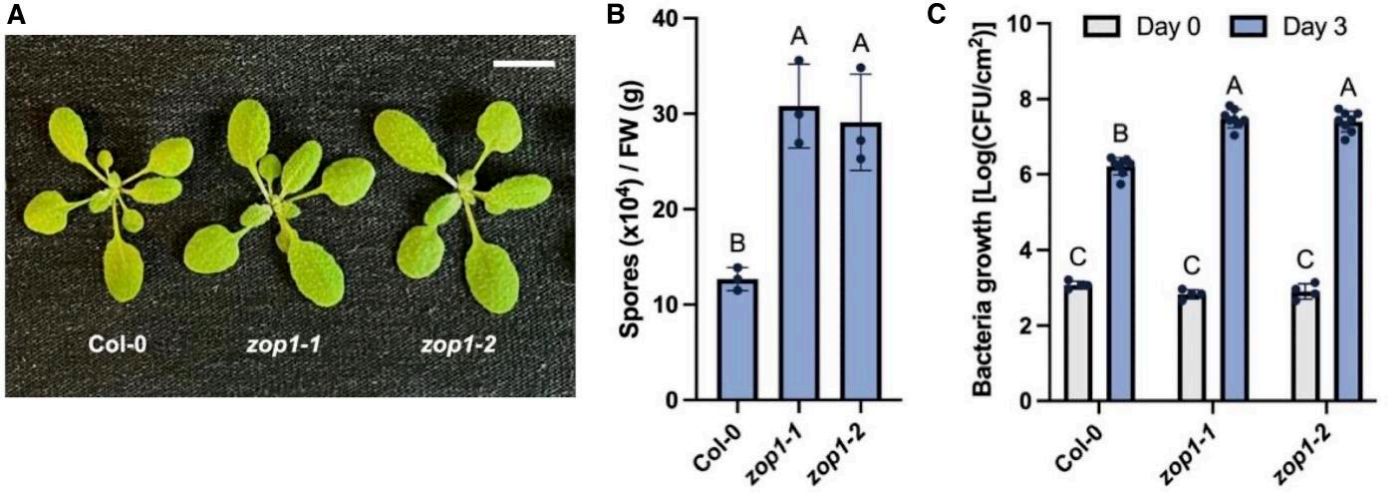
Compromised immunity in the *zop1* mutants. **A)** Morphology of 21-day-old soil-grown plants of the indicated genotypes under long-day conditions. Scale bar is 1 cm. **B)** Growth of *Hpa* Noco2 conidiospores on the indicated genotypes. Error bars represent standard deviations. Letters indicate statistical differences (*P* < 0.05, one-way ANOVA; *n* = 3). FW, fresh weight. **C)** Growth of *Pst* DC3000 on the indicated genotypes. Error bars represent standard deviations. Letters indicate statistical differences (*P* < 0.0001, one-way ANOVA; *n* = 4 for Day 0, *n* = 8 for Day 3). CFU, colony-forming units. All experiments were repeated three times with similar results.

To study the role of ZOP1 in plant immunity, *zop1* single mutants were generated in Col-0 background using CRISPR/Cas9. Two independent *zop1* deletion alleles, *zop1-1* and *zop1-2*, show similar stature as wild-type plants (Fig. 3A and Supplementary Fig. S5). To determine whether disease resistance is affected in the *zop1* mutants, oomycete pathogen *Hpa* Noco2 and bacterial pathogen *Pst* DC3000 were inoculated on wild-type plants and the *zop1* mutants. As shown in Fig. 3, B and C, *zop1* mutants supported significantly more *Hpa* Noco2 and *Pst* DC3000 growth than wild-type plants, indicative of a positive role of ZOP1 in plant immunity.

### *zop1* single mutant has decreased *BDA1* transcript level

**Figure 4.**
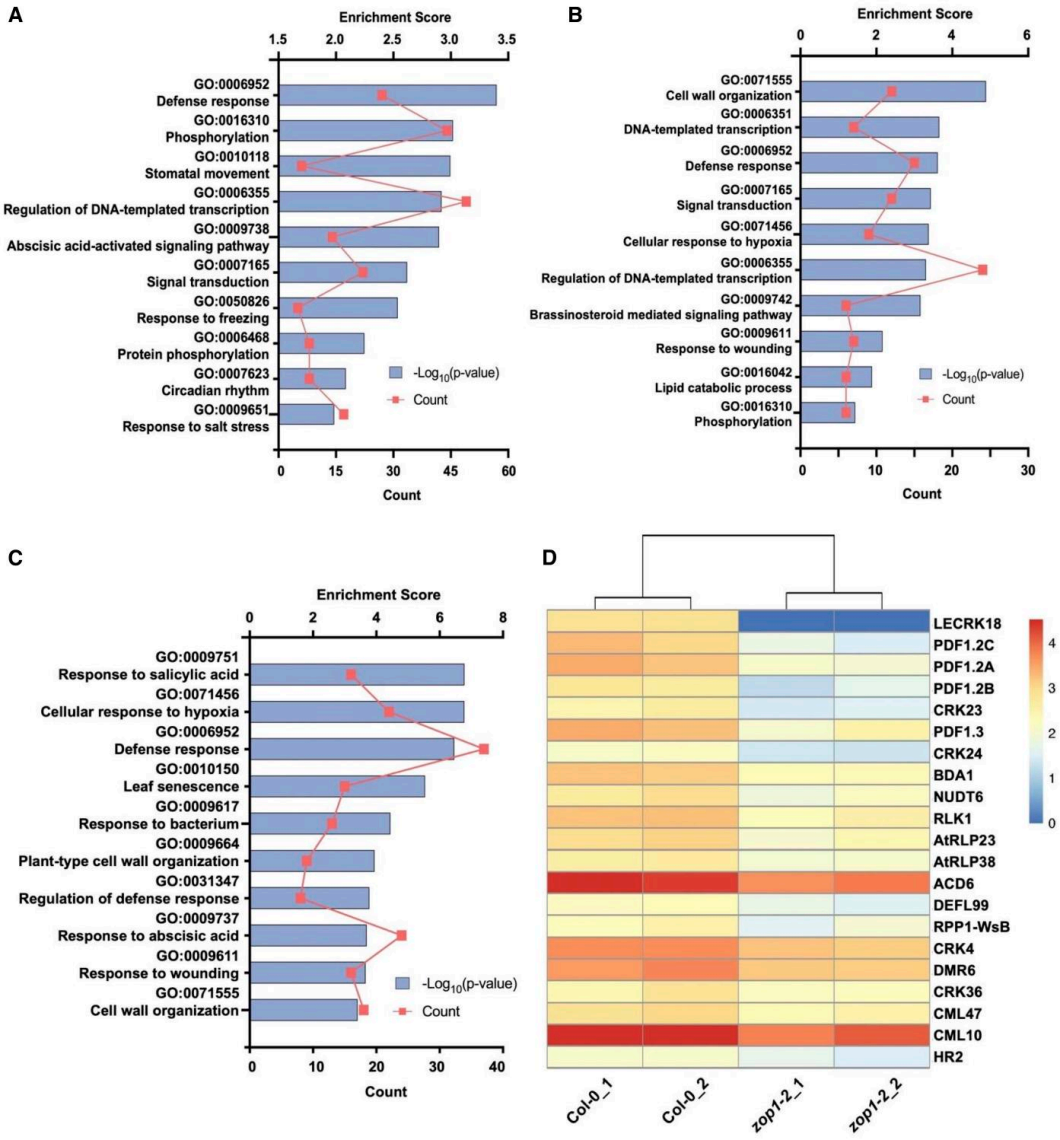
Analysis of RNA-Seq data from the wild-type and *zop1* mutant plants. **A** to **C)** GO enrichment analysis of the biological functions of 428 alternatively spliced genes **(A)**, 338 upregulated genes **(B)**, and 763 downregulated genes **(C)** in *zop1-2* compared to the wild type. The top 10 enriched GO terms ranked by enrichment scores are shown in bar graph. The plot graph indicates the number of downregulated genes in each GO term. **D)** Heatmap visualization of downregulated defense-related genes in *zop1-2* compared to wild-type. Each row of the heatmap represents a gene, each column represents a sample, and each cell displays normalized gene expression values. Blue color represents low expression and red color represents high expression.

Since ZOP1 was known to be involved in alternative splicing and transcriptional silencing (Zhang et al. 2013), RNA-sequencing (RNA-seq) was carried out on wild-type and *zop1-2* mutant plants. We examined both alternatively spliced genes and differentially expressed genes in the *zop1-2* mutant. Using replicate Multivariate Analysis of Transcript Splicing (rMATS) approach, 377 intron-retention events in 350 genes, 25 alternative 3′ splicing sites in 22 genes, 38 alternative 5′ splicing sites in 32 genes and 24 genes with skipped exons were identified in *zop1-2* mutant (adjusted *P*-value < 0.01). Transcripts of 763 genes showed over 2-fold reduction and 338 genes showed over 2-fold increase in *zop1-2* mutant compared to wild-type plant. Gene ontology (GO) analysis of the biological functions of the alternatively spliced and differentially expressed genes revealed that genes related to defense responses are enriched (Fig. 4A-4C), suggesting a critical role of ZOP1 in plant immunity. However, we did not find any obvious alternatively spliced genes that can explain the suppression of *snc2-1D*-mediated autoimmunity. In contrast, from the list of the differentially expressed genes, we noticed that the transcript level of *BDA1*, which encodes an essential regulator of SNC2-mediated immunity (Yang et al. 2012), was downregulated in the *zop1-2* mutant (Fig. 4D).

**Figure 5.**
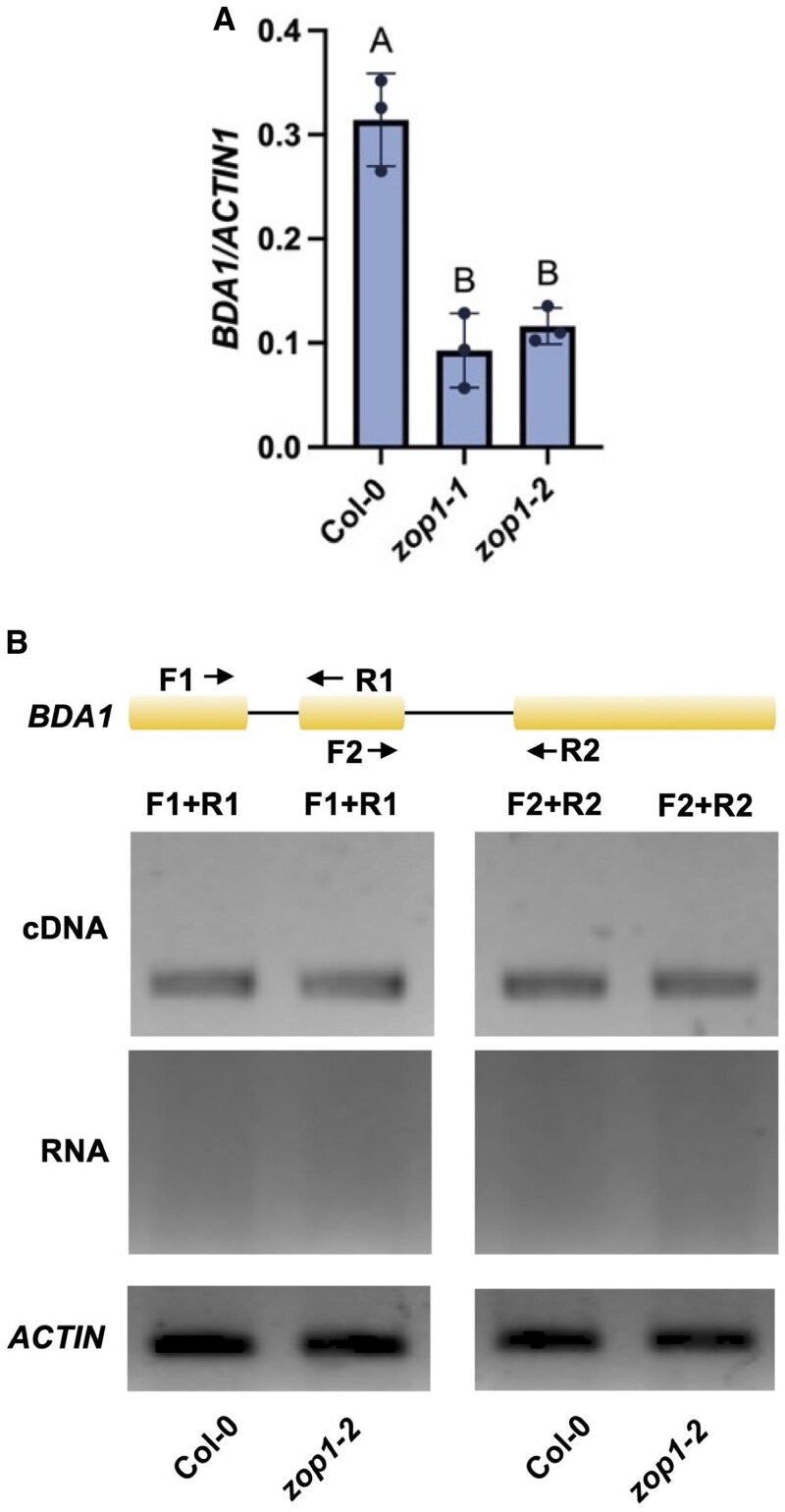
Analysis of the transcript level and splicing pattern of *BDA1* in the *zop1* mutants. **A)** Expression levels of *BDA1* in the indicated genotypes as normalized by *ACTIN1*. Error bars represent standard deviations. Letters indicate statistical differences (*P* < 0.05, one-way ANOVA; *n* = 3). **B)** Splicing analyses of *BDA1* for the *zop1-2* mutant. PCR was performed on cDNA or RNA samples under identical conditions. cDNA samples used in the PCRs were normalized using *ACTIN1*. All experiments were repeated three times with similar results.

To confirm the RNA-seq data, reverse transcription quantitative PCR (RT-qPCR) analysis was performed on wild-type and *zop1* mutant plants. As shown in Fig. 5A, transcript level of *BDA1* was decreased in *zop1* single mutants. Also, decreased *BDA1* transcript levels were detected in the suppressor 116-1 and CRISPR *ZOP1* lines in *sard1-1 scn2-1D* (Supplementary Fig. S6). Although ZOP1 is known to contribute to splicing, no intron-retention event was detected in *BDA1*. PCR using primers flanking introns of *BDA1* did not detect any alternatively spliced transcripts between wild-type and *zop1-2* either (Fig. 5B). Thus, ZOP1 seems only to affect the transcript level of *BDA1* but not its pre-mRNA splicing process.

As deletion of *ZOP1* was able to suppress the induced *CBP60g* expression in *sard1-1 scn2-1D* (Fig. 2E), the transcript level and splicing pattern of *CBP60g* were also analyzed in *zop1* single mutant. Surprisingly, neither the transcript level nor the splicing of *CBP60g* was affected by *zop1* mutation (Supplementary Fig. S7). The reason might be because the regulation of CBP60g is intricate, it includes but not limited to the transcriptional regulation from transcription factors and the post-transcriptional regulation from calmodulins and calcium-dependent protein kinases (Huang et al. 2020). Loss of *zop1* may lead to decreased activity of both unknown negative and positive regulators of CBP60g and ultimately maintain the wild-type-like transcript level of *CBP60g* in *zop1* single mutant.

### *zop1* single mutant exhibits attenuated nlp20-induced immunity

**Figure 6.**
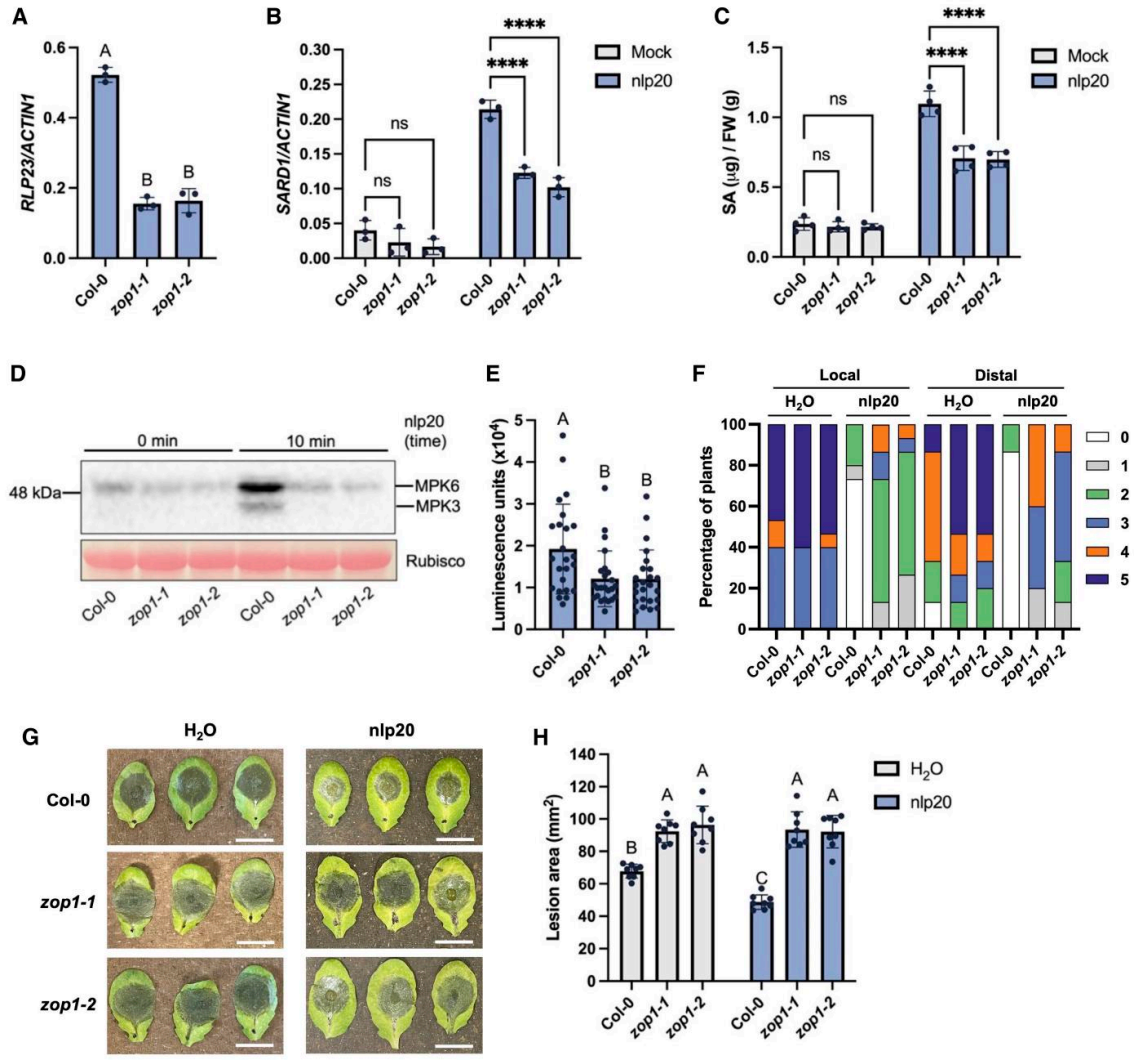
*RLP23* expression and nlp20-induced immune responses in *zop1* single mutants. **A)** Expression levels of *RLP23* in the indicated genotypes as normalized by *ACTIN1*. Error bars represent standard deviations. Letters indicate statistical differences (*P* < 0.05, one-way ANOVA; *n* = 3). **B)** Relative expression levels of *SARD1* in the indicated genotypes treated with H_2_O (mock) or 1 *μ*m nlp20 for 4 h. Values were normalized to the expression of *ACTIN1*. Error bars represent standard deviations. **** indicates statistical differences (*P* < 0.0001, one-way ANOVA; *n* = 3). **C)** SA levels in the indicated genotypes treated with H_2_O (mock) or 1 *μ*m nlp20 for 24 h. Error bars represent standard deviations. **** indicates statistical differences (*P* < 0.0001, one-way ANOVA; *n* = 4). FW, fresh weight. **D)** nlp20-induced MAPK activation in the indicated genotypes. 12-day-old seedlings grown on ½ MS medium plates were sprayed with 0.1 *μ*m nlp20 and samples were harvested at 10 min. Total proteins from each sample were extracted for western blot analysis with anti-p44/42-ERK antibody. Equal loading is shown by Ponceau S staining of a nonspecific band. **E)** nlp20-induced ROS production in the indicated genotypes measured as luminescence. The values show the peak of luminescence units through all the time points. Error bars represent standard deviations (*P* < 0.05, one-way ANOVA; *n* = 24 leaf disks from different plants). **F)** Growth of *Hpa* Noco2 on the local or distal leaves of the indicated genotypes with or without nlp20 treatment. Plants were pretreated with H_2_O or 1 *μ*m nlp20 and sprayed with *Hpa* Noco2 spores (5 × 10^4^ spores/mL) 24 h later. Fifteen plants were used for each treatment. Disease ratings (0 to 5) showing the relative growth of *Hpa* Noco2 are described in the methods. 0: no conidiophores on plants; 5: more than twenty conidiophores on two infected leaves or more than two distal leaves are infected with more than five conidiophores. **G)** Growth of *B. cinerea* on the indicated genotypes. Plants were pretreated with H_2_O or 1 *μ*m nlp20 and inoculated with 10 *μ*L *B. cinerea* conidial suspensions (10^5^ conidia/mL) 24 h later. Representative photographs were taken at 48 h post inoculation. The scale bar is 1 cm. **H)** Quantification of lesion sizes in **(G)**. The dots represent the values of lesion areas measured by ImageJ. Error bars represent standard deviations. Letters indicate statistical differences (*P* < 0.0001, one-way ANOVA; *n* = 8). All experiments were repeated twice with similar results.

Among the genes downregulated in *zop1-2* identified by RNA-seq analysis, *RLP23* encodes a receptor-like protein which specifically recognizes a 20-amino-acid motif (nlp20) of NECROSIS- AND ETHYLENE-INDUCING PEPTIDE 1-LIKE PROTEINS in microorganisms and contributes to the activation of downstream immune signaling (Böhm et al. 2014; Albert et al. 2015). RT-qPCR analysis confirmed that *zop1* single mutants exhibit decreased transcript levels of *RLP23* (Fig. 6A). Therefore, immune responses induced by nlp20 were examined in wild-type plant and *zop1* single mutants. As shown in Fig. 6B, the expression level of *SARD1* after nlp20 treatment was reduced in *zop1* single mutants comparing to that in wild-type plant. Accordingly, SA level after nlp20 treatment was lower in the *zop1* single mutant (Fig. 6C). In addition, as shown in Fig. 6, D and E, nlp20 treatment induced strong MITOGEN-ACTIVATED PROTEIN KINASE (MAPK) activation and reactive oxygen species (ROS) burst in wild-type but not *zop1* single mutant plants. Furthermore, nlp20-induced local and systemic resistance to *Hpa* Noco2 was impaired in the *zop1* single mutants (Fig. 6F). The nlp20 primed wild-type plants with enhanced immunity to infection by the fungal pathogen *Botrytis cinerea*, while the lesion sizes in *zop1* single mutants were significantly larger (Fig. 6, G and H). Overall, loss of *ZOP1* affects immune responses induced by nlp20.

Since ZOP1 regulates immunity mediated by both RLP SNC2 and RLP23, we further explored whether ZOP1 exerts a broader role in PTI. Wild-type and *zop1* mutant plants were treated with flg22, a short peptide of the bacterial flagellin protein that induces PTI (Gómez-Gómez and Boller 2000). Upon flg22 elicitation, *zop1* mutants were able to induce comparable levels of MAPK phosphorylation, ROS burst and upregulation of *FLG22-INDUCED RECEPTOR-LIKE KINASE 1* (*FRK1*) as wild-type plants (Supplementary Fig. S8), suggesting that flg22-induced defense responses were not affected in *zop1* mutants. Therefore, ZOP1 does not seem to broadly regulate PTI.

## Discussion

**Figure 7.**
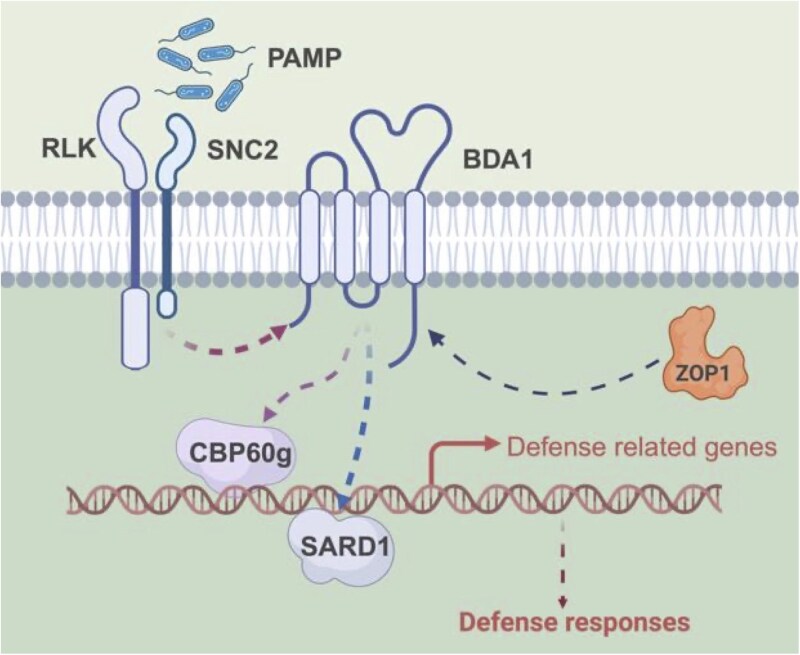
A working model of ZOP1 in SNC2-mediated immunity. The splicing factor ZOP1 positively impacts SNC2-mediated immunity by modulating the transcript level of *BDA1*.

From a suppressor screen of *sard1-1 snc2-1D*, we found that the pre-mRNA splicing factor ZOP1 is required for SNC2-mediated immunity. Mutations in *ZOP1* partially suppress the autoimmune phenotype of *sard1-1 snc2-1D*, including the elevated *PR* and *CBP60g* gene expression, increased SA accumulation and enhanced disease resistance (Figs. 1 to 2). In addition, loss of *ZOP1* suppresses the dwarf stature of *snc2-1D* and *cbp60g-1 snc2-1D* (Fig. 2H and Supplementary Fig. S4), indicating that ZOP1 functions downstream of SNC2 before defense signals branch to CBP60g and SARD1 (Fig. 7). Moreover, *zop1* single mutants exhibit compromised basal resistance against bacterial pathogens (Fig. 3), and many defense-related genes are alternatively spliced or differentially expressed in *zop1* mutants (Figs. 4 to 6). Thus, ZOP1 plays an important role in plant immunity regulation in addition to regulating pre-mRNA splicing, transcriptional gene silencing and cold stress responses.

ZOP1 is a pre-mRNA splicing factor that associates with components of the splicing machinery including STA1 and RDM16 (Du et al. 2015). It is required for the expression of *BDA1*, which encodes an essential positive regulator for SNC2-mediated immunity. However, the effect of ZOP1 on *BDA1* expression does not appear to be through altering its pre-mRNA splicing (Fig. 4), suggesting that BDA1 is unlikely a direct target of ZOP1. One possibility is that ZOP1 contributes to the alternative splicing of a transcription factor, which positively regulate *BDA1* transcription, and alteration of its splicing results in the reduced *BDA1* transcript level in *zop1* mutants. Alternatively, ZOP1 may regulate *BDA1* transcription through transcriptionally silencing of a negative regulator of *BDA1* transcription. What is the direct regulator(s) of *BDA1* needs further investigation.

In *Arabidopsis*, the perception of PAMP nlp20 and subsequent activation of immune responses require the PRR RLP23, whose transcript level is regulated by different transcription factors, e.g. WRKY8 and CBP60g (Kim et al. 2022; Ren et al. 2024). Our transcriptome analysis and RT-qPCR result showed that the pre-mRNA splicing factor ZOP1 also regulate the expression of *RLP23*, where the effect seems to be at the transcript level only, as no intron resides within the *RLP23*. As a splicing factor, the effect of ZOP1 on *RLP23* transcription is unlikely to be direct. How exactly ZOP1 regulates the transcripts of these plant immunity components awaits further analysis.

GO term enrichment analysis on the *zop1-2* mutant revealed that genes responsive to SA are also differentially expressed in the *zop1* mutant. The regulation of ZOP1 does not seem to be limited to SA-dependent immune responses, with RLP23 as an example (Fig. 6). Furthermore, among those differentially expressed genes in the *zop1-2* mutant, some encode defense responses-related cysteine-rich receptor-like kinases (CRKs). *Arabidopsis* CRKs form a subgroup of RLKs with more than 40 members (Chen 2001). Their extracellular domains contain 2 copies of the conserved Cys-X8-Cys-X2-Cys motif. In the past few decades, some CRKs were demonstrated to play critical roles in disease resistance in *Arabidopsis*. For example, overexpression of *CRK4* and *CRK36* leads to enhanced PTI responses (Yeh et al. 2015). As RLPs lack a cytoplasmic kinase domain, they were proposed to associate with RLKs to transduce defense signal to downstream components (Couto and Zipfel 2016). Whether these CRKs participate in SNC2-mediated immunity would be interesting to examine in the future.

## Materials and methods

### Plant materials and growth conditions

All *Arabidopsis* (*Arabidopsis thaliana)* plants used in this study are in the Col-0 ecotype. The *sard1-1 snc2-1D* mutants were reported previously (Sun et al. 2015). *zop1-1* and *zop1-2* are 2 independent deletion lines generated by CRISPR/Cas9. Plants used in this study were grown on soil under a long-day condition of 22 °C and 16 h light/8 h dark cycles unless specified. Plants used for SA quantification, *Pst* DC3000 infection, nlp20-induced SAR assay, and *B. cinerea* infection were grown on soil under a short-day condition of 8/16-h light/dark regime at 22 °C.

### Construction of plasmids

To generate the CRISPR/Cas9 constructs for knocking out *ZOP1*, genomic sequence of *ZOP1* was subjected to CRISPR-PLANT (http://www.genome.arizona.edu/crispr/CRISPRsearch.html) to select the guide RNAs. The selected guide RNAs were evaluated with Cas-OFFinder (http://www.rgenome.net/cas-offinder/). The *pHEE401E* vector was used to generate the CRISPR/Cas9 constructs according to previously published protocols (Wang et al. 2015).

For transgene complementation, the genomic DNA fragment of *ZOP1* driven by its native promoter was PCR-amplified from wild-type genomic DNA and cloned into pBasta-3HA vector to obtain *pZOP1::ZOP1*. All primers used are listed in Supplementary Table S1.

### Mutant screening

Ethyl methane sulfonate (EMS) mutagenesis was performed following a previously described protocol (Li and Zhang 2016). In brief, around 5,000 *sard1-1 snc2-1D* mutant seeds were mutagenized, sterilized, and planted on ½ Murashige and Skoog (½MS) medium. Ten-day-old well established seedlings (M1 generation) were transplanted onto soil and grown to maturity. About 40,000 M2 progeny from 2,000 M1 plants were grown on soil and screened for plants with increased plant size compared with the *sard1-1 snc2-1D* mutant.

### Mapping-by-sequencing

Mapping-by-sequencing was performed as previously described (Sun et al. 2020). The *116-1* mutant was backcrossed with *sard1-1 snc2-1D* plant. 39 of total 169 F2 segregants that showed larger size were kept to F3 generation. Leaf tissue from 36 confirmed larger homozygous plants were pooled together and used for DNA extraction. The resulting genomic DNA was sequenced by Illumina WGS. The frequency of SNPs was calculated using Excel and used to identify the linkage region and candidate mutations.

### SA quantification

For SA quantification, plants were grown on soil under a short-day condition for 4 weeks. SA extraction and measurement was performed as a previously described procedure (Li et al. 1999). For each sample, around 100 mg leaf tissue was collected from 3 individual plants of the indicated genotypes and ground into fine powder with liquid nitrogen. Plant tissue was resuspended with 600 *μ*L 90% methanol and sonicated for 20 min to release SA. After centrifugation at 12,000 × *g* for 10 min, the supernatant was collected, and the pellets underwent a second round of extraction by adding 500 *μ*L of 100% methanol and sonicating for another 20 min. The supernatant from both extractions were combined and dried by vacuum. About 500 *μ*L 5% (w/v) trichloroacetic acid was added to the dry samples, vortexed, and sonicated for 5 min. The samples were subsequently centrifuged at 12,000 × *g* for 15 min. The supernatant was collected and then extracted 3 times with 500 *μ*L extraction buffer (ethylacetate acid/cyclopentane/isopropanol at 100:99:1 by volume). Each time, after centrifugation at 12,000 × *g* for 1 min, the organic phase was collected and combined to a new tube and dried by vacuum afterwards. The final dried sample was resuspended in 500 *μ*L mobile phase (0.2 m KAc, 0.5 mm EDTA pH = 5) by vortexing and sonication for 5 min. After spinning at 12,000 × *g* for 5 min, the supernatant was kept and analyzed by high-performance liquid chromatography to measure the amount of SA as compared with a standard.

### Pathogen infection assay

For *Hpa* Noco2 infection, 2-week-old seedlings were sprayed with conidiospores suspension at a concentration of 5 × 10^4^ spores per mL water. Plants were subsequently covered with a transparent lid and transferred to a growth chamber at 18 °C under a 12 h day/12 h night cycle with 80% humidity. Sporulation was quantified at 7 days post inoculation by counting conidia spores with a hemocytometer. Four to six individual plants were pooled as a single sample.

For *Pst* DC3000 infection assays, plants were grown on soil under a short-day condition for 4 weeks. The fifth and sixth leaves of plant were infiltrated with *Pst* DC3000 suspension at a dose of OD_600_ = 0.0002 in 10 mm MgCl_2_ with a needleless syringe. Bacterial growth was scored on Days 0 and 3. Two leaf discs from each of the inoculated leaves on the same plant were collected as one sample. Samples were ground in 10 mm MgCl_2_ and diluted serially with 10 mm MgCl_2_. Bacteria colonies from selected dilution were counted after 36 h of incubation at 28 °C and calculated as colony-forming unit (cfu).

For nlp20-induced SAR assays, plants were grown on soil under a short-day condition for 3 weeks. Two primary leaves of plants were first infiltrated with 1 *μ*m nlp20 or H_2_O. After 24 h, plants were sprayed with *Hpa* Noco2 spore suspension at a concentration of 5 × 10^4^ spores per mL water. Then plants were covered with a transparent lid and transferred to a growth chamber at 18 °C under a 12 h day/12 h night cycle with 80% humidity. Infection was scored at 7 days post inoculation by counting the number of conidiophores per infected leaf. A total of 15 plants were scored for each treatment. For infection on the local leaves, disease rating scores are as follows: 0: no conidiophores on the infected leaves; 1: no more than five conidiophores on one infected leaf; 2: six to twenty conidiophores on one infected leaf; 3: twenty or more conidiophores on one infected leaf; 4: five or more conidiophores on two infected leaves; 5: twenty or more conidiophores on two infected leaves. For infection on the distal leaves, disease rating scores are as follows: 0: no conidiophores on plants; 1: one leaf is infected with no more than five conidiophores; 2: one leaf is infected with more than five conidiophores; 3: two leaves are infected but with no more than five conidiophores on each infected leaf; 4: two leaves are infected with more than five conidiophores on each infected leaf; 5: more than two leaves are infected with more than five conidiophores.

For *B. cinerea* infection, plants were grown on soil under a short-day condition for 4 weeks. Leaves of plants were first infiltrated with 1 *μ*m nlp20 or H_2_O. After 24 h, infiltrated leaves were detached from the plant and drop-inoculated with 10 *μ*L droplets of *B. cinerea* isolate B05.10 containing 10^5^ spores per mL in potato dextrose broth (Shanghai Bio-Way Technology). Inoculated leaves were placed on moistened paper towels in a container covered with lids to maintain humidity and continuous darkness at 23 °C. Photographs were taken 48 h after infection and lesion sizes were determined using ImageJ software.

### MAPK activation assay

*Arabidopsis* seedlings for MAPK activation assay were grown on ½ MS medium under long-day condition for 12 days. Seedlings were sprayed with 0.1 *μ*m nlp20 or H_2_O. Six seedlings for each treatment were collected afterwards at 0 and 10 min and then ground into fine powder with liquid nitrogen. Protein was extracted from these seedlings with 2 × SDS loading buffer, and the phosphorylation of MAPKs was detected by immunoblots with anti-p44/42-ERK antibody (Cell Signaling; #9102; 1:2,500 dilution). Loading consistency was examined by staining the membrane with Ponceau S.

### Measurement of ROS production

Leaves of 4-week-old short-day-grown plants were sliced into strips and submerged in water in a 6-well plate for around 16 h, with the adaxial side facing upward. The leaves were then transferred into 1.5 mL microcentrifuge tubes and treated with 0.1 *μ*m flg22 or 1 *μ*m nlp20 in 200 *μ*L solution containing 20 *μ*m luminol and 1 *μ*g/mL peroxidase. Luminescence readings were collected immediately with an integration time of 60 s using a GloMax 20/20 luminometer (Promega, Canada). The peak of luminescence units measured was used as a measurement of ROS production.

### Gene expression and splicing pattern analysis

*Arabidopsis* seedlings for RNA extraction were grown on ½ MS medium under long-day condition for 12 days. Each sample consists of RNA from about 6 seedlings. RNA was extracted using the Plant RNA Mini-Preps Kit (Bio Basic, Canada) and treated with RQ1 RNAase-free DNAase (Promega, Canada) to eliminate genomic DNA contamination. Reverse transcription reactions were carried out with OneScript Reverse Transcriptase (Abcam, Canada). Quantitative RT-qPCR was performed using the SYBR Premix Ex Taq II (Takara, Japan). Primers used for amplification of *PR1*, *PR2*, *SARD1*, *FRK1* and *ACTIN1* were described previously (Zhang et al. 2003; Sun et al. 2015; Sun et al. 2018). The primers for analyzing the transcription level and the intron-retention events of *BDA1* and *CBP60g* are included in Supplementary Table S1.

### RNA-seq analysis

For RNA-seq, 12-day-old seedlings of wild-type and *zop1-2* grown on ½ MS media were collected. RNA extraction and RNA-seq were carried out using services provided by Smartgenomics Technology Institute (Tianjin, China). Two independent RNA samples from each genotype were mixed prior to library preparation and sequencing. For quality, 39–62 million raw reads were checked, and low-quality reads, adapter sequences, and contaminations were filtered out. The clean reads of each sample were aligned to the publicly available reference genome of *Arabidopsis* (TAIR10) using Hierarchical Indexing for Spliced Alignment of Transcripts 2 (HISAT2) (Kim et al. 2019). Transcript assembly and quantification were performed using StringTie (Pertea et al. 2015). GO analysis was completed using the statistical enrichment test from the Database for Annotation, Visualization and Integrated Discovery (DAVID, https://david.ncifcrf.gov/). For intron-retention analysis, rMATS tool was used to identify reliable expressed introns and adjusted *P*-value < 0.05 were regarded as intron-retention events (Shen et al. 2014).

### Statistical analysis

Error bars in all figures represent standard deviations. The number of biological replicates is indicated in the figure legends. The statistical significance of the data was determined using GraphPad Prism 10 software (GraphPad Software Inc., San Diego, CA, USA: http://www.graphpad.com/). Statistical comparison among different samples is carried out by one-way ANOVA with Tukey's honestly significant difference post hoc test as shown in the figure legends.

### Accession numbers

The accession numbers for the sequence data from this article can be found from the National Center for Biotechnology Information (NCBI, https://www.ncbi.nlm.nih.gov/) or GenBank: ZOP1 (AT1G49590), BDA1 (AT5G54610), and RLP23 (AT2G32680). The RNA-Seq data have been submitted to the National Center for Biotechnology Information Sequence Read Archive (http://www.ncbi.nlm.nih.gov/sra) under BioProject accession PRJNA1188206.

## Supplementary Material

kiaf351_Supplementary_Data

## Data Availability

The data underlying this article are available in the article and in its online Supplementary material.

## Supplementary data

The following materials are available in the online version of this article.

**Supplementary Figure S1.** Mapping-by-sequencing of 116-1

**Supplementary Figure S2.** ZOP1 deletion mutations in *sard1-1 snc2-1D* generated by CRISPR/Cas9.

**Supplementary Figure S3.**  *pZOP1::ZOP1* complements the defects of the suppressor 116-1.

**Supplementary Figure S4.**  *zop1-2* mutation suppresses the dwarfism of *cbp60g-1 snc2-1D*.

**Supplementary Figure S5.**  *ZOP1* deletion mutations in Col-0 background generated by CRISPR/Cas9.

**Supplementary Figure S6.** Suppressed expression of *BDA1* in *ZOP1* CRISPR deletion lines in *sard1-1 snc2-1D*.

**Supplementary Figure S7.** Analysis of the transcript level and splicing pattern of *CBP60g* in the *zop1* mutants.

**Supplementary Figure S8.** flg22-induced immune responses were not affected in *zop1* mutants.

**Supplementary Table S1.** Primers used in this study.
