# Supplementary material for: The pre-mRNA splicing factor ZOP1 contributes to the regulation of plant immunity
Source: Plant Physiol. 2025 Aug 6;199(1):kiaf351. doi: 10.1093/plphys/kiaf351 (PMC12406697; doi:10.1093/plphys/kiaf351)
Supplement: kiaf351_Supplementary_Data [file kiaf351_supplementary_data.pdf]

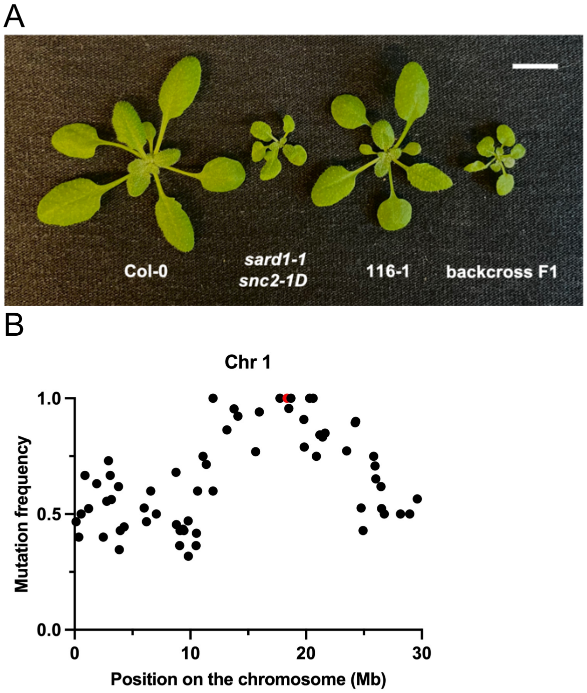

Supplementary Figure S1. Mapping-by-sequencing of 116-1.

(A) Morphologies of 24-day-old soil-grown plants of the indicated genotypes under long-day condition. Scale bar is 1 cm. (B) Linkage map showing the linkage region of the 116-1 mutation on chromosome 1. The mutation in *ZOP1* is highlighted using a red data point.

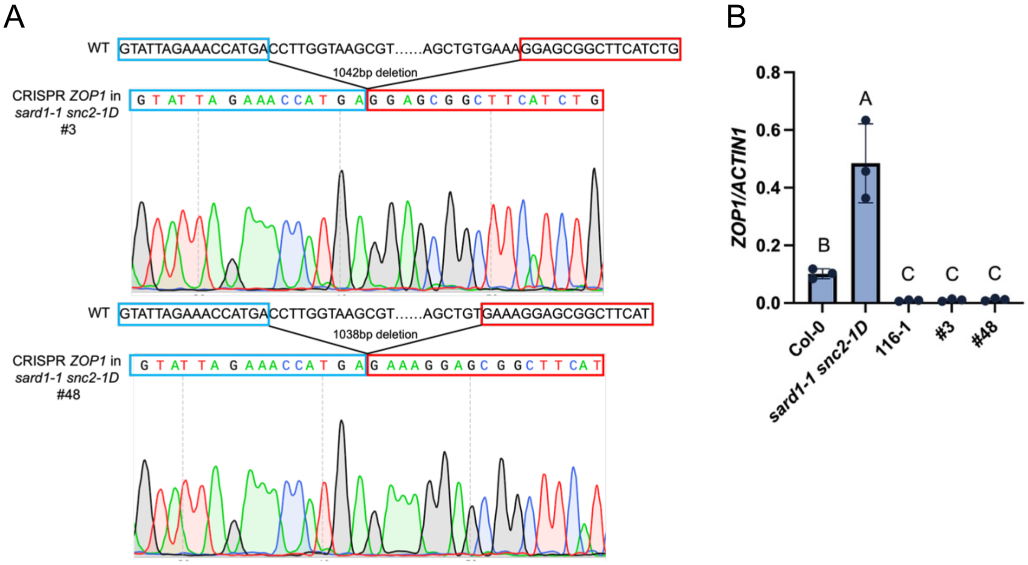

Supplementary Figure S2. *ZOP1* deletion mutations in *sard1-1 snc2-1D* generated by CRISPR/Cas9.

(A) Sequences of deletion mutations in *ZOP1* generated by CRISPR/Cas9. (B) Expression levels of *ZOP1* in the indicated genotypes as normalized by *ACTIN1*. Error bars represent standard deviations. Letters indicate statistical differences ($P < 0.05$, one-way ANOVA; $n = 3$). Experiments were repeated twice with similar results.

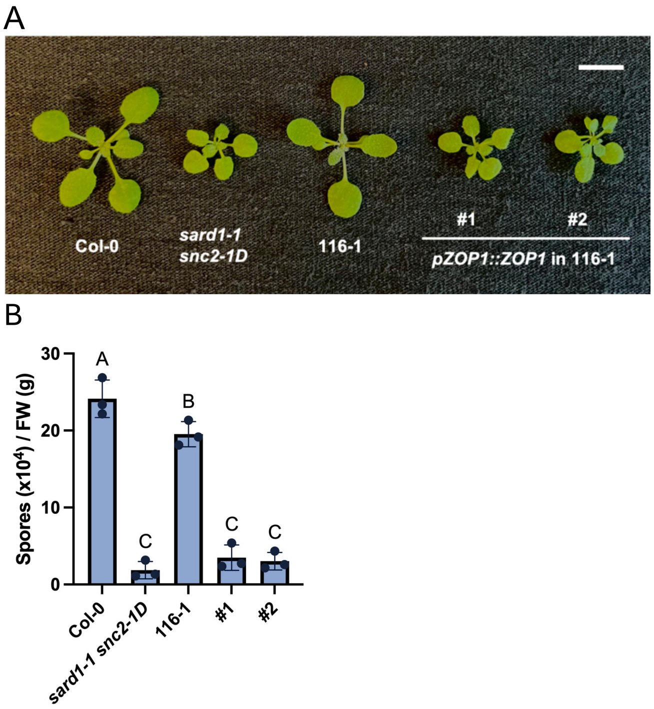

Supplementary Figure S3. ***pZOP1::ZOP1*** **complements the defects of the suppressor 116-1.**

(A) Morphology of 21-day-old soil-grown plants of the indicated genotypes under long-day condition. #1 and #2 are two independent complementation lines of *ZOP1* in the 116-1 background. Scale bar is 1 cm. (B) Growth of *Hpa* Noco2 conidiospores on the indicated genotypes. Error bars represent standard deviations. Letters indicate statistical differences ($P < 0.05$, one-way ANOVA; $n = 3$). FW, fresh weight. Experiments were repeated twice with similar results.

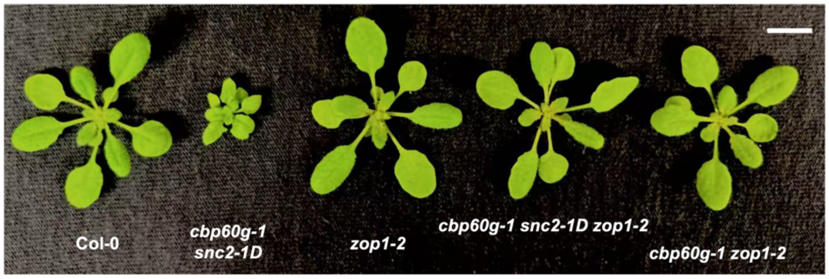

Supplementary Figure S4. ***zop1-2*** **mutation suppresses the dwarfism of** ***cbp60g-1 snc2-1D.***

Morphologies of 24-day-old soil-grown plants of the indicated genotypes under long-day condition. Scale bar is 1 cm.

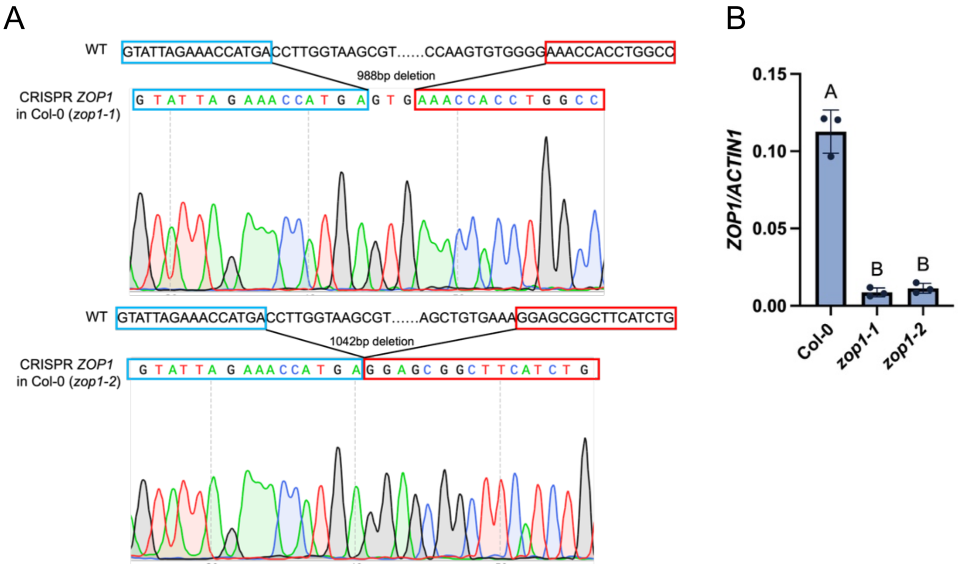

Supplementary Figure S5. *ZOP1* deletion mutations in Col-0 background generated by CRISPR/Cas9.

(A) Sequences of deletion mutations in *ZOP1* generated by CRISPR/Cas9. (B) Expression levels of *ZOP1* in the indicated genotypes as normalized by *ACTIN1*. Error bars represent standard deviations. Letters indicate statistical differences (P < 0.05, one-way ANOVA; n = 3). Experiments were repeated twice with similar results.

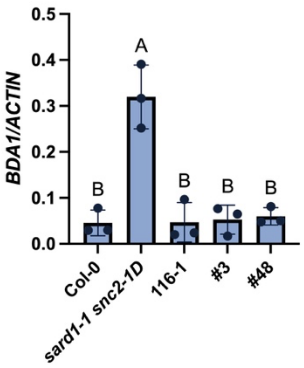

Supplementary Figure S6. Suppressed expression of *BDA1* in *ZOP1* CRISPR deletion lines in *sard1-1 snc2-1D*.

Expression levels of *BDA1* in the indicated genotypes as normalized by *ACTIN1*. Error bars represent standard deviations. Letters indicate statistical differences (P < 0.05, one-way ANOVA; n = 3). Experiments were repeated twice with similar results.

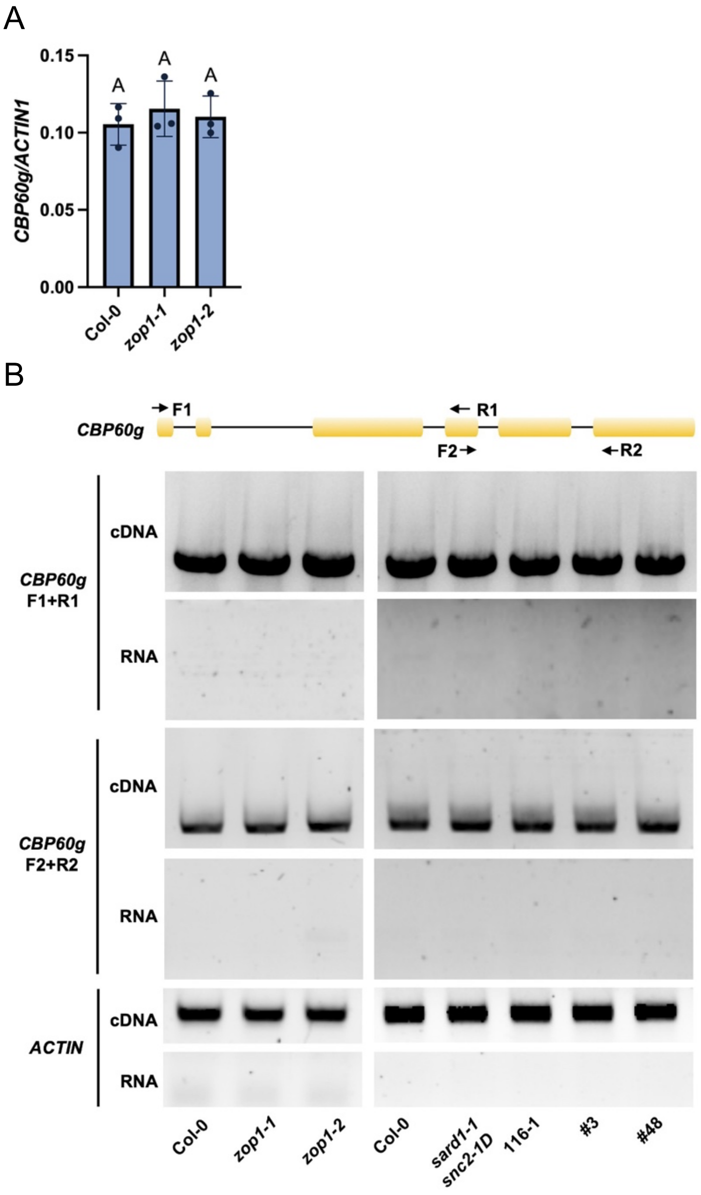

Supplementary Figure S7. Analysis of the transcript level and splicing pattern of *CBP60g* in the *zop1* mutants. (A) Expression levels of *CBP60g* in the indicated genotypes as normalized by *ACTIN1*. Error bars represent standard deviations. Letters indicate statistical differences ($P < 0.05$, one-way ANOVA; $n = 3$). (B) Splicing analyses of *CBP60g* in the indicated genotypes. PCR was performed on cDNA or RNA samples under identical conditions. cDNA samples used in the PCR reactions were normalized using *ACTIN1*. All experiments were repeated twice with similar results.

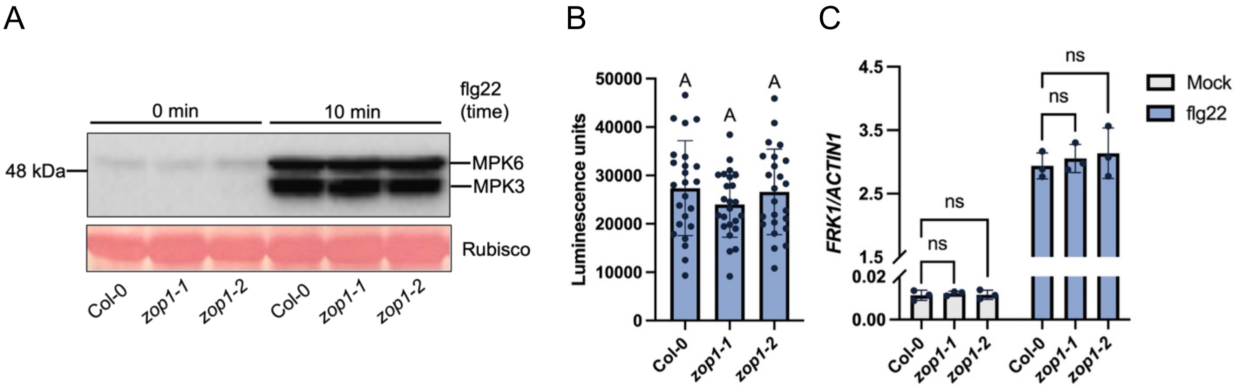

Supplementary Figure S8. flg22-induced immune responses were not affected in *zop1* mutants.

(A) flg22-induced MAPK activation in the indicated genotypes. 12-day-old seedlings grown on ½ MS medium plates were sprayed with 0.1 μM flg22 and samples were harvested at 10 min. Total proteins from each sample were extracted for western blot analysis with anti-p44/42-ERK antibody. Equal loading is shown by Ponceau S staining of a non-specific band. (B) flg22-induced ROS production in the indicated genotypes measured as luminescence. The values show the peak of luminescence units through all the time points. Error bars represent standard deviations ($P < 0.05$, one-way ANOVA; n = 24 leaf disks from different plants). (C) Relative expression levels of *FRK1* in the indicated genotypes treated with H_2O (mock) or 1 μM flg22 for 4 h. Values were normalized to the expression of *ACTIN1*. Error bars represent standard deviations. ns indicates no statistical differences ($P < 0.05$, one way-ANOVA; n = 3). All experiments were repeated twice with similar results.

Supplementary Table S1. Primers used in this study.

Primer name	Primer sequence (5' to 3')
At1g49590-DT1-BsaI-F	ATATATGGTCTCGATTGGTATTAGAAACCATGACCTGTTTTAGAG CTAGAAATAGC
At1g49590-DT2-BsaI-R	ATTATTGGTCTCGAAACCTCCTTTCACAGCTCTTTTCAATCTCTTA GTCGACTCTAC
At1g49590-dele-F	TTCGCATCTTTTCTCTCCAAA
At1g49590-dele-R	AGCAGCTTCTCTTGCCTTCA
At1g49590-homo-F	GGTGCACCAGAGGATGGTA
At1g49590-pro-KpnI-F	CCGGGGTACCCAGAGAATGACGCAAAACCA
At1g49590-SpeI-R	CGGCTAGACTAGTAAAGGGTCTATTGTAAAGAC
At1g49590-RT-F	TGCACTATGATTCACAGTCTGG
At1g49590-RT-R	ACACTTGGTCCAGCTCCTG
BDA1-RT-F	CGTGGTAGAGGAGGTATGACAC
BDA1-RT-R	GCATCCACCCTGTGAGAACT
BDA1-splicing-F1	CAGAAAATGCGCGATAGTGA
BDA1-splicing-R1	AGAGAGCCTCCGGTCTTACC
BDA1-splicing-F2	TTTGGCAATGGAACTGATGA
BDA1-splicing-R2	TCACTATCGCGCATTTTCTG
RLP23-RT-F	TCCCCTGTCTATGGCCAATC
RLP23-RT-R	CCGTGAGTTGGTTGTGAGAC
CBP60g-splicing-F1	ATGAAGATTCGGAACAGCCCTA
CBP60g-splicing-R1	GCACGGAGGATGATGTTTTT
CBP60g-splicing-F2	GAAAATCGCGAAAGATGGAG
CBP60g-splicing-R2	GAAGCTGTTTCCGAAAGTCG
